# Characterization of the complete mitochondrial genome of the coral reef fish, *Hemigymnus melapterus* (Pisces: Labridae) and its phylogenetic implications

**DOI:** 10.1080/23802359.2019.1693302

**Published:** 2019-11-20

**Authors:** Murong Yi, Sui Gu, Zhisen Luo, Hung-Du Lin, Yunrong Yan

**Affiliations:** a College of Fisheries, Guangdong Ocean University, Zhanjiang, China;; b Marine Resources Big Data Center of South China Sea, Southern Marine Science and Engineering Guangdong Laboratory, Zhanjiang, China;; c The Affiliated School of National Tainan First Senior High School, Tainan, Taiwan;; d Guangdong Provincial Engineering and Technology Research Center of Far Sea Fisheries Management and Fishing of South China Sea, Guangdong Ocean University, Zhanjiang, China;; e Center of Marine Fisheries Information Technology, Shenzhen institute of Guangdong Ocean University, Shenzhen, China

**Keywords:** *Hemigymnus melapterus*, mitochondrial genome, next-generation sequencing

## Abstract

*Hemigymnus melapterus* belongs to the family Labridae, which inhabit in coastal and continental shelf waters. The entire mitochondrial genome of *H. melapterus* is 16,527 base pairs (bp) in length and contained 13 protein-coding genes, two rRNA genes, and 22 tRNA genes. The overall base composition is 27.56% A, 25.58% T, 30.02% C, and 16.85% G, showing AT-rich feature (53.14%). Phylogenetic analysis based on 13 protein-coding genes shows the *H. melapterus* has the closest evolutionary relationship with *Stethojulis strigiventer*. This work provides valuable genome variation information, which will be useful for phylogenetic analysis and population genetics research.

## Mitogenome announcement

Coral reefs are exceptionally diverse ecosystems and there are over 4000 species of fish associated with coral reefs (Cowman et al. 2009). The family Labridae containing 82 genera and about 600 species of fishes is the fifth largest fish family and second largest marine fish family. Labrids inhabit coastal and continental shelf waters in tropical and temperate oceans throughout the world, especially in the Coral reefs (Westneat and Alfaro 2005). *Hemigymnus melapterus* was found in subtidal reef flats and lagoon and seaward reefs. This is the first study to determine the complete mitochondrial genome of *H. melapterus* for the first time and explored the phylogenetic relationship among Labridae fish (24 species in total) available in GenBank complete mitochondrial genomic database, contributing to the molecular evolution and phylogenetic relationship of this species with members of Labridae and further protection of its genetic resources.

Samples of *H. melapterus* were collected from Haikou of Hainan Island (E 110.5334, N 20.1255) in China. The entire specimen was stored in 95% ethanol and registered in the College of Fisheries, Guangdong Ocean University under the voucher number GOU101613. Genomic DNA extraction and next-generation sequencing were described in previous publication (Chiu et al. 2018). Whole-genome Shotgun DNA library was constructed and sequenced by Illumina HiSeq platform (Illumina, CA, USA). Totally, it produced 21,917,444 clean reads with a size of 3,634,660,800 base pairs (bp). The complete mitogenome of *H. melapterus* was assembled with A5-miseq v20150522 (Coil et al. 2015) and SPAdes (Bankevich et al. 2012). Protein-coding genes, ribosomal RNA genes and transfer RNA genes were identified using MITOS (Bernt et al. 2013) tool and manually inspected.

The size of *H. melapterus* mitochondrial genome was 16,527 bp (GenBank Accession Number: MN614148), containing 25.58% of T, 30.02% of C, 27.56% of A, and 16.85% of G. It harbored 13 protein-coding genes (PCGs), 22 transfer RNAs (tRNAs), two ribosomal RNAs (12S and 16S rRNAs), and a D-loop control region. All protein-coding genes start with ATG, except for COI and ATP6, which used GTG as the start codon. The stop codon of seven protein-coding genes (ND1, COI, ATP8, ATP6, COIII, ND4L, and ND6) is TAA, whereas the ND2 and ND5 genes use TAG and AGG as the stop codon, respectively. The remaining protein-coding genes (COII, ND3, ND4, and Cyt *b*) have incomplete stop codons T.

**Figure 1.**
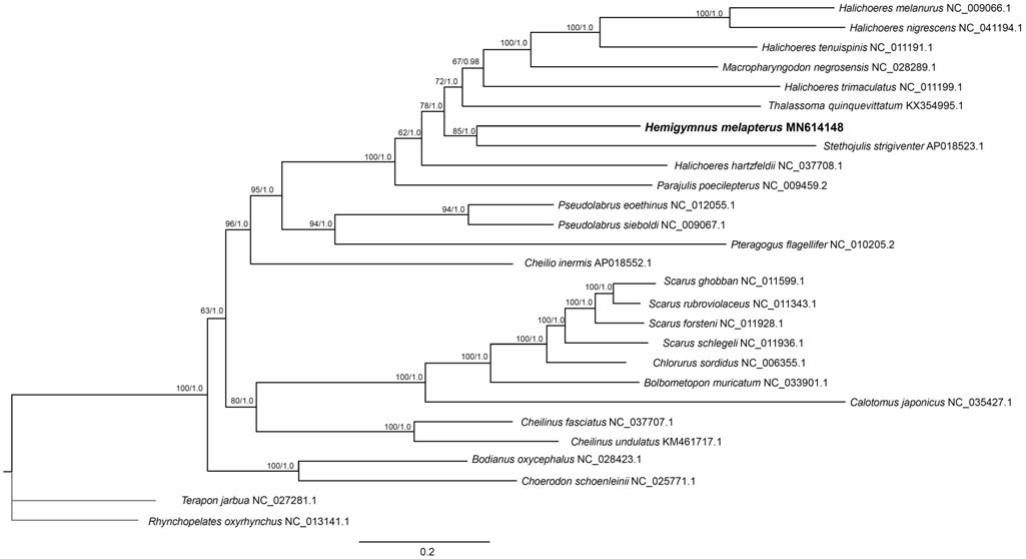
Phylogenetic tree of *Hemigymnus melapteru* and 24 related species in Labridae based on 13 concatenated mitochondrial protein-coding genes by maximum likelihood (ML) and Bayesian (BA) analysis. The numbers on the nodes are the bootstrap values from ML/BA analyses. The mitochondrial genome sequence from *Terapon jarbua* (GenBank Accession Number: NC_027281.1) and *Rhynchopelates oxyrhynchus* (GenBank Accession Number: NC_013141.1) were used as outgroup.

To determine the phylogenetic position of *H. melapterus*, phylogenetic trees were reconstructed by maximum likelihood (ML) and Bayesian (BA) methods based on the 13 PCGs, implemented in PhyloSuite (Zhang et al. 2019). Molecular phylogenetic analysis reveals based on the mitogenome sequences that *H. melapterus* has the closest evolutionary relationship with *Stethojulis strigiventer* (Figure 1). They are grouped together as sister groups, which is similar to previous research based on partial mitochondrial sequences and nuclear genes (Westneat and Alfaro 2005). For further evaluation of the phylogenetic relationships within the family Labridae, more complete mitogenome information that encompasses the taxonomic diversity of the family is required.
